# Sunlight and Health

**Published:** 1891-07

**Authors:** 


### SUNLIGHT AND HEALTH.

This is what Florence Nightingale says of the value of light to those who are ill, no less than for those who are well. " Second only to fresh air, I should be inclined to rank light. Direct sunlight, not only daylight, is necessary for speedy recovery." Instances could be given, almost endless, where in dark wards, or in wards with a northern aspect, even when thoroughly warmed, or in wards with borrowed light, even when thoroughly ventilated, the sick could not by any means be made speedily to recover. The dark side of the street is far more subject to disease than the light side. Sir James Wilie found three times as many cases of disease on the shaded side of the barracks at St. Petersburg as on the other side. A prominent physician, Dr. J. N. Farrar, who has paid special attention to the effect of the presence or absence of light in living rooms upon health, found that, in his own case, when occupying a room facing north, his general health was not nearly so good as when his window had a southern exposure. General experience will confirm this conclusion. Human beings, like plants, need an abundance of light, and, if denied it, they pine and wilt.

